# Impact of DSA and immunosuppression minimization on rejection, graft, and patient survival after simultaneous liver–kidney transplantation

**DOI:** 10.3389/fmed.2022.949833

**Published:** 2022-08-22

**Authors:** Manon Dekeyser, Jean-Luc Taupin, Michelle Elias, Philippe Ichaï, Florence Herr, Marc Boudon, Melanie Brunel, Antonio Sa cunha, Audrey Coilly, Faouzi Saliba, Antoine Durrbach

**Affiliations:** ^1^Nephrology and Transplantation Department, APHP, Henri Mondor Hospital, Créteil, France; ^2^INSERM UMR 1186, Institut Gustave Roussy, Villejuif, France; ^3^Paris-Saclay University, Paris, France; ^4^HLA Laboratory, AP-HP Saint Louis Hospital, Paris, France; ^5^University of Paris, Paris, France; ^6^APHP, Paul Brousse Hospital, INSERM UMR 1193, Villejuif, France

**Keywords:** donor-specific antibody, simultaneous liver–kidney transplantation, acute rejection (AR), drug minimization, liver transplant, kidney transplantation

## Abstract

**Background:**

Acute rejection rate is low after simultaneous liver–kidney transplantation (SLKT), leading some groups to minimize immunosuppressive (IS) regimens. However, the impact of preformed (pDSA) or *de novo* donor-specific antibodies (dnDSA) on the graft remains unclear.

**Methods:**

We performed a retrospective analysis of 102 consecutive SLKT patients to study the impact of anti-HLA antibodies.

**Results:**

Anti-HLA antibodies were detected in 75 recipients (class I 23.8%, both classes I and II 23.8%, and class II 14.3%). In total, 42.8% of the patients had pDSA and 21.7% developed dnDSA. Overall patient survival at 1–3 and 5 years, was respectively 88, 84, and 80%. Acute rejection occurred respectively in 3 (2.9%) liver and 6 kidney (5.9%) recipients. pDSA with titers over 10,000 mean fluorescence intensity (14.3%) was associated with lower patient survival (40 vs. 82%) but not with acute rejection. In a multivariable Cox regression analysis, the risk of death was associated with maleness, the highest titer of pDSA (*p* < 0.0007) or the sum of pDSA >10,000. Renal function did not differ between patients with class I pDSA (*p* = 0.631) and those with class II pDSA (*p* = 0.112) or between patients with and without a positive cross-match (*p* = 0.842). dnDSA were not associated with acute rejection, graft dysfunction or patient survival. IS minimization was not associated with rejection, graft dysfunction or death.

**Conclusion:**

In SLKT, high levels of pDSA >10,000 were associated with lower patient survival, but not rejection or graft survival. Minimization of maintenance immunosuppression regimen was not associated with a poorer outcome.

## Introduction

Allogenic immune responses leading to graft rejection are a major challenge in solid organ transplantation. The rate of graft loss has been reduced by cross-matching to detect the presence of pre-existing anti-HLA antibodies and the use of a combination of immunosuppressive drugs to control the activation of allogeneic T cells. Interestingly, organs differ in their global immunogenicity, with some more able to initiate T-cell activation than others, such as the liver, which is more tolerant. Different immunosuppressive regimens are therefore required for different organs. In addition, combined liver–kidney transplantation has been shown to be associated with a lower risk of acute rejection (1).

Over the last decade, antibodies against donor-specific antibody (DSA) have emerged as a major factor of graft loss following solid organ transplantation (2–4). The presence of low levels of preformed donor-specific antibodies (pDSAs), which can be detected with sensitive solid-phase assays, increases the risk of antibody-mediated rejection (AMR) and allograft failure in kidney transplantation (2–4). pDSA can bind to endothelial cells, inducing endothelial injury through complement-dependent cytotoxicity (CDC) or antibody-dependent cell-mediated cytotoxicity (ADCC), and stimulating cell proliferation (5, 6). The resulting lesions are characterized by the inflammation of peritubular or glomerular microvessels (7). By contrast, liver allografts are relatively resistant to AMR and many transplant centers do not check for the presence of pDSA at time of transplantation. Spontaneous liver tolerance appears to develop in approximately 20% of cases (8, 9). The mechanisms underlying this apparent tolerance are not fully understood but may be dependent on the large mass of the liver, the ability of hepatocytes to regenerate, and the exposure of liver cells to antigens and microbial products from the gastrointestinal tract, resulting in a unique immunological microenvironment. Recent studies have suggested that only high titers of DSA affect the outcome of liver transplantation (8, 10, 11).

In simultaneous liver–kidney transplantation (SLKT), the two grafts behave differently. The liver has been reported to protect the kidney against hyperacute rejection, even in the presence of high levels of DSA possibly by reducing the level of both class 1 and class 2 circulating DSA following the liver implantation (12–14). Before the advent of Luminex assay technology, we and others reported a lower incidence of acute and chronic rejection in cases of SLKT than in cases of kidney transplantation alone (KTA) (1, 15–17), suggesting that it might be possible to minimize the maintenance immunosuppressive regimen. However, recent data suggest that preformed and *de novo* DSA (dnDSA) can induce acute and chronic graft dysfunction and accelerate graft fibrosis and biliary strictures in liver transplantation alone (LTA) (15). Moreover, various forms of kidney rejection associated with poorer graft function have been reported in up to 20% of SLKT patients (18, 19). O’Leary et al. showed that only class II pDSA are associated with poorer patient and graft outcomes (20, 21).

We describe here our experience over the last 10 years, since the advent of Luminex technology for SLKT, and we evaluate the impact of DSA and of minimizing maintenance immunosuppression on patient’s liver and kidney outcomes.

## Materials and methods

### Study population

All patients who underwent SLKT at our institution between January 2008 and December 2018 were included retrospectively. A pre-SLKT serum sample was available for all patients, for the detection of single antigens and CDC cross-matching. Deceased-donor transplants were matched for ABO compatibility. Data were obtained from the French national registry of the “Agence de la Biomédecine,” and from electronic clinical, laboratory, and pathology reports. All recipients received induction therapy with either basiliximab (Novartis^®^) or polyclonal antithymoglobulins (ATG) (Genzyme^®^) and intravenous methylprednisolone, followed by a triple immunosuppressive regimen combining prednisone, tacrolimus, and mycophenolate mofetil.

The maintenance regimen included tacrolimus (8–10 ng/ml for the three first months and 4–8 ng/ml thereafter). Mycophenolate mofetil (2 g/day) was decreased stepwisely after month 6 every 2 months. The decrease was interrupted in case of acute rejection. Prednisone was decreased in a stepwise manner. Other immunosuppressive agents, such as cyclosporine and everolimus, were also used, but in only a small proportion of patients. This study was approved by the Institutional Review Board of Paris-Sud University.

### Immunological analyses

Recipient and donor human leukocyte antigen (HLA) typing were performed using SSO (low resolution between 2008 and 2015, high resolution between 2015 and 2016) (LabType reagents, One Lambda, Canoga Park, CA, United States) or NGS (since 2017, GenDX reagents, Utrecht, Netherlands) for recipients, and serology (mainly One Lambda) or low-resolution SSP (mainly Olerup, Stockholm, Sweden), or intermediate resolution (mainly SABR, Linkage Biosciences, Thermo Fisher, Waltham, MA, United States) for donors depending on their geographic origin. Loci A, B, DRB1, and DQB1 were typed at minimum, with in addition Cw and DQA1 in SSO, and also DPB1 and DRB3/4/5 in SABR and NGS. T-cell and B-cell CDC cross-matching was performed at the time of transplantation using the standard method (i.e., without anti-human globulin enhancement step), looking for IgG and IgM cytotoxic DSA. Only IgG positive crossmatches were considered as positive crossmatches in the present work. No autologous crossmatches were performed. The presence and specificity of anti-HLA antibodies were assessed prospectively on recipient serum samples collected before transplantation, with LABScreen (One Lambda, Canoga Park, CA, United States) reagents LSM12 for the screening step, and LS1A04 and LS2A01 for class I and II single-antigen beads on a Luminex platform. Pre-transplant, screening was performed first and followed by SAFB assay in both classes when found positive in at least one class. Post-transplant, only single antigen assays were performed. Anti-HLA antibody levels were expressed as mean fluorescence intensity (MFI) values after normalization of the non-specific binding using the Baseline formula of the Fusion^®^ (One Lambda) software. A baseline MFI >500 was considered positive.

### Liver and kidney function

Liver allograft loss was defined as liver retransplantation or death from liver failure. Estimated glomerular filtration rate (eGFR) was calculated with the original chronic kidney disease epidemiology (CKD-EPI) equation [eGFR = 141 × min (SCr/κ, 1)^α^ × max(SCr/j, 1)^–1.209^ × 0.993^Age^ × 1.018 (if female) × 1.159 (if black)]. Kidney allograft loss was defined as kidney retransplantation or a need for renal replacement therapy.

### Liver and kidney allograft biopsies

Liver and kidney biopsies were performed to evaluate allograft dysfunction following an increase in serum liver enzyme levels or a 25% decrease in eGFR, respectively (for-cause biopsies). Protocol biopsies were performed only for liver allografts, 1 and 5 years after transplantation. Liver and kidney biopsy specimens displaying signs of rejection were analyzed and scored according to the Banff classification (22, 23).

### Statistical analysis

The results are presented as absolute values (percentages) or as the median (range). Categorical variables were analyzed in Fisher’s exact tests. Continuous variables were compared in Mann–Whitney *U* tests. The impact of pDSA on patient survival and graft outcomes was analyzed by the Kaplan–Meier method, with log-rank tests. Time-to-event analysis was performed for death and graft failure, with censoring at the last known visit. Statistical analyses were performed with JMP software. A *p*-value < 0.05 was considered statistically significant.

## Results

### Demographic and transplant characteristics

In total, 102 patients underwent SLKT at our institution between January 2008 and December 2018. Their baseline characteristics are displayed in Table 1. Their mean age was 50 ± 13.4 years, and 40% of the patients were female. The SLKT was a repeat transplantation in 34% of patients: 25% underwent retransplantation of the liver, and 8% underwent retransplantation of the kidney. The three leading causes of transplantation were viral cirrhosis, alcoholic cirrhosis and polycystic kidney-liver disease for the liver, and glomerulonephritis, calcineurin inhibitor toxicity and polycystic kidney disease for the kidney. None of the patients had a refractory hepatorenal syndrome.

**TABLE 1:** Patient characteristics.

Recipient	
Age (mean ± SD, year)	50 ± 13.4
Sex: female (%)	40 (40%)
Ethnicity, Caucasian (%)	84 (82%)
BMI (mean ± SD, kg/m^2^)	23.5 ± 5.6
Prior liver transplant (%)	25.5
Prior kidney transplant (%)	8.4
MELD score (mean ± SD)	22.7 ± 8.9
Liver disease *n* (%)	
Viral hepatitis	22 (21.5%)
Alcohol-induced cirrhosis	16 (15.7%)
Metabolic syndrome	3 (2.9%)
Amylosis	7 (6.8%)
Primary oxaluria	4 (3.9%)
Autosomic polycystic kidney disease	20 (19.6%)
Biliary atresia	3 (2.9%)
Autoimmune cirrhosis	5 (4.9%)
Hepatocellular carcinoma	7 (6.8%)
Re-transplantation and other	15 (14.7%)
Kidney disease *n* (%)	
Glomerulonephritis	24 (25.5%)
Polycystic kidney disease	23 (22.5%)
Vascular disease	3 (2.9%)
Diabetes mellitus	5 (4.9%)
Interstitial nephropathy	8 (6.1%)
CNI toxicity	15 (14.7%)
Primary oxaluria	4 (3.9%)
Unknown	18 (17.6%)
Transplant	
Donor age (mean ± SD, year)	49.9 ± 13.3
Induction therapy %	
None	16.6
IL-2R antagonist	54.2
T-cell depletion	29.2
Maintenance therapy at initiation *n* (%)	
Calcineurin inhibitors	
Tacrolimus	101 (96%)
Cyclosporine A	4 (4%)
Mycophenolate mofetil	100 (95.2%)
Steroids	105 (100%)
Maintenance therapy at 1 year *n* (%)	
Calcineurin inhibitors	86 (96.6%)
Tacrolimus	81 (91%)
Cyclosporine	5 (5.6%)
Tacrolimus trough level (ng/ml)	7.8 (0–13.2)
Mycophenolate mofetil therapy	47 (52%)
Mycophenolate mofetil dose (mg/day)	500 (0–2,000)
Steroids	67 (75%)
Steroid dose (mg/day)	5 (0–16)
mTOR inhibitor	4 (4.5%)
Triple regimen	37 (43%)
Double regimen	44 (51.2%)
Single drug	5 (5.8%)

Anti-HLA antibodies were detected with a MFI >500 in 75% of patients (*n* = 75), and these patients were considered sensitized (Table 2). At the time of transplantation, anti-HLA antibody levels exceeded 1,000 MFI units in 56% of patients. The antibodies were directed against class I only in 24 patients, both classes I and II in 23.8% of patients and class II only in 14.3% of patients (Table 3). The proportions of women and retransplanted patients were higher among the sensitized than among the non-sensitized patients.

**TABLE 2:** Patient characteristics, by DSA absence/presence.

	Non-immunized patients no HLA antibodies *N* = 27	Sensitized patients with pHLA antibodies (+) *N* = 75	Sensitized patients with pDSA *N* = 43
Recipient			
Age (year)	49.1 ± 13.15	50.4 ± 14.1	51.1 ± 11.9
Sex: female (percentage)	23.5%	51.5%	52.4%
BMI	23.5 ± 5.1	23.4 ± 6	24.7 ± 1
Prior liver	26%	27%	43%
transplant			
Prior kidney transplant	3%	11%	10%
Death rate	23.8%	17.2%	20%
Early death or re-transplantation	23.8%	17.2%	20%
Early re-transplantation	4.76%	3.45%	0%
Biliary complication	4.1%	15.4%	6%
Transplant			
Donor age (year)	51 ± 13.1	49.1 ± 13.7	45.2 ± 15.8
Positive cross-match (LCT)	12.2%	10.8%	0%
Induction therapy			
None	9.6	21.4	18.2
IL-2R antagonist	80.9	35.7	24.2
T-cell depletion	9.5	42.9	57.6
Graft function			
at 1 year			
eGFR at 1 year	49.4 ± 21.1	55.1 ± 18.6	57.4 ± 18.5
Proteinuria at 1 year	2.7 ± 1	0.18 ± 0.16	1.11 ± 1.4
ALT at 1 year	38.9 ± 37.2	32.3 ± 28.3	34.5 ± 29.4
Gamma-GT at 1 year	132 ± 183.5	72.7 ± 75.3	131.9 ± 203.8
Rejection			
Kidney, *n* = 5	7.4%	4.1%	6.9%
Liver, *n* = 3	3.7%	2.7%	2.3%
Maintenance therapy at 1 year			
Calcineurin inhibitors	100%	100%	89.3%
Tacrolimus	90.7	96.4	73.6
Cyclosporine A	10.3	3.6	15.8
Mycophenolate mofetil	38%	63.6%	15.8
Steroids	68.9%	81.8%	68.3
mTOR inhibitor	3.4%	5.4%	5.2
Triple regimen	20.7%	54.6%	19%
Double regimen	62.1%	45.4%	68%
Single drug	17.2%	0%	13%

**TABLE 3:** Anti-HLA antibodies in SLKT patients.

	%
Preformed anti-HLA (%)	
Yes	55.6
No	44.4
Class I	23.8
Class II	14.3
Classes I + II	23.8
pDSA (%)	
Yes	42.85
No	57.15
Class I	13.1
Class II	21.4
Classes I + II	8.3
MFI DSA >5,000	15.4
MFI DSA >10,000	5.9
Total DSA MFI >10,000	14.3
Cross-match (LCT) (%)	
Positive (IgM + IgG)	10.1
IgG	4
Cross-match or pDSA	49.6
pDSA still present at month 1 (%)	
Yes	18.6
No	81.4
Class I	5.3
Class II	9.3
Class I + II	4
*De novo* DSA (%)	
Yes	21.7
No	78.3
Class I	2.9
Class II	18.6
Classes I + II	0
MFI >1,000	6.9
Maximum MFI	3607

Preformed donor specific antibody were detected in 42.8% of the patients, against class I only in 13.1%, against both classes I and II in 8.3%, and against class II only in 21.4% (Table 2). Taking the MFI of pDSA into account, 15.4% of the patients had a highest pDSA with a MFI >5,000, and 5.9% had a highest pDSA with a MFI >10,000. Some patients had several pDSA, and 14.3% of the patients had a total MFI for all the pDSA present that exceeded 10,000. At transplantation, 10% of the patients had a positive CDC cross-match (IgM and IgG) and 4% had an IgG-positive cross-match. The type of induction regimen differed significantly (*p* < 0.003) according to immunological status. Non-sensitized patients were more frequently prescribed rIL2-blocking antibodies, whereas sensitized patients and patients with pDSA were more frequently prescribed polyclonal depleting antibodies. All patients with a positive cross-match received in addition, polyvalent immunoglobulins (IVIG) at the time of transplantation. In all but one of these patients, the cross-match became negative after liver implantation. The patient in whom the cross-match persisted after liver implantation, albeit at lower intensity, was treated by plasma exchange followed by IVIG, rituximab, and eculizumab for 6 months. We also detected a positive cross-match in three pDSA-negative patients.

One month after transplantation, 18.6% of patients still had pDSA, with the same HLA family distribution as before transplantation, but with a lower MFI (Table 3). pDSA were reduced by 56.6% 1 month after transplantation. The decrease was similar for class 1 and class 2 pDSA respectively, 59.5 and 56.5%. For both HLA classes, pDSA disappearance was independent of their pre transplant MFI titers and were independent of the different loci of HLA molecules (i.e., A/B/C/DR/DQ).

### Overall patient survival

**FIGURE 1:**
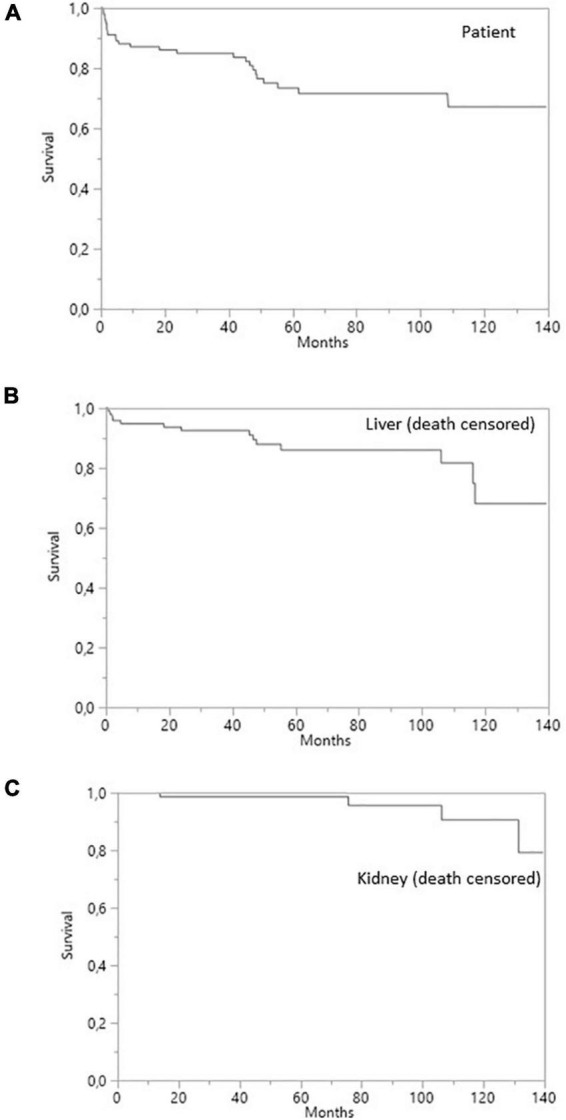
Survival curves for SLKT patients. **(A)** Patient survival. **(B)** Liver survival (death-censored). **(C)** Kidney survival (death-censored).

**FIGURE 2:**
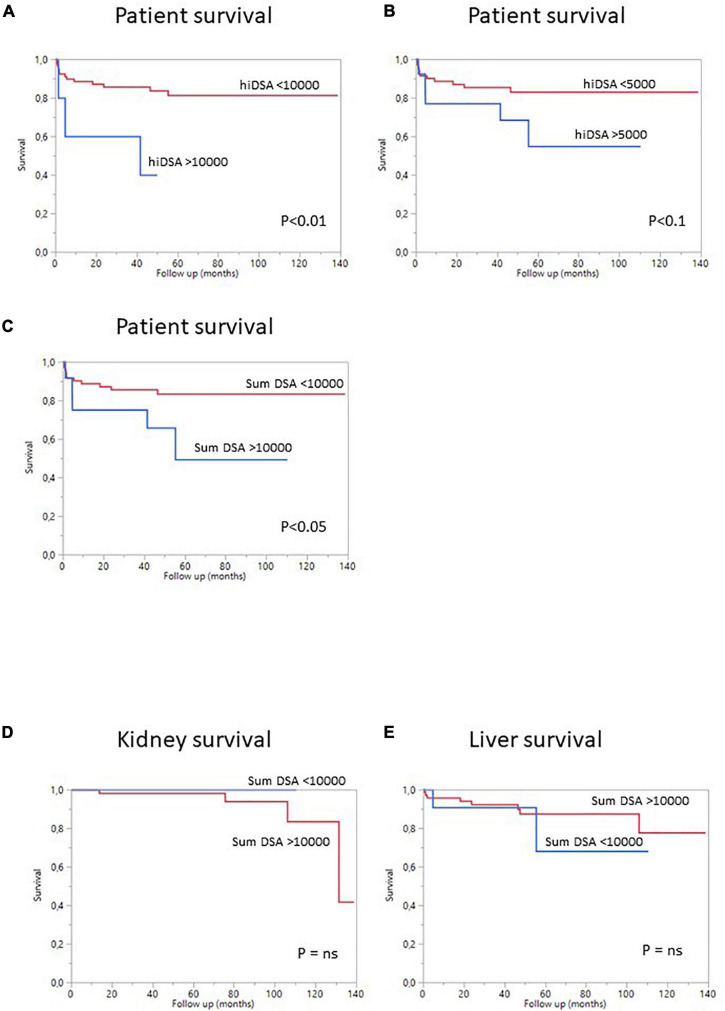
Survival curves for SLKT patients according to pDSA status. **(A)** Survival curves for patients with a highest pDSA with a MFI >10,000 (blue) or a highest pDSA with a MFI <10,000 MFI (red). **(B)** Survival curves for patients with a highest pDSA with a MFI >5,000 MFI (blue) or a highest pDSA with a MFI <5,000 (red). **(C)** Survival curves for patients with a MFI for total pDSA >10,000 MFI (blue) or a MFI for total pDSA <10,000 MFI (red). **(D)** Death-censored kidney survival in patients with a highest pDSA with a MFI >10,000 (blue) or <10,000 (red). **(E)** Death-censored liver survival in patients with a highest pDSA with a MFI >10,000 (blue) or <10,000 MFI (red).

Overall patient survival was 88% at 1 year, 84% at 3 years, and 80% at 5 years (Figure 1). The leading cause of death was infection [*n* = 14 patients (13.8%)]. The other causes of death were cardiovascular diseases [*n* = 4 (3.9%)], and *de novo* cancer (*n* = 1) (Table 4). The risk of death was not associated with Model for End-Stage Liver Disease (MELD) score at the time of transplantation (*p* = 0.084) but was associated with being male (*p* < 0.001), serum creatinine concentration at 1 year (*p* = 0.0339), and gamma glutamine transferase (GGT) levels at 1 year (*p* = 0.0015) (Table 5). The risk of death was not associated with the presence of pDSA or a positive cross-match, but it was associated with the presence of a highest pDSA with a MFI >10,000 (*p* < 0.037) and a sum of MFI for all pDSA >10,000 (*p* < 0.05) (Figures 2A,C), but not for highest pDSA > 5000 (Figure 2B). The HLA loci or the class against which pDSA is directed has no impact on the outcome. We found that early death (within 3 months) and early retransplantation (within 1 month) for the liver, mostly due to primary graft non-function, were associated only with the rank of liver transplantation (*p* < 0.0359) and not with a positive cross-match or the presence of pDSA (Table 5). In a multivariate analysis, it was associated with the sex (*p* < 0.04) and tend to be associated with the highest pDSA >10,000 (Table 6).

**TABLE 4:** Cause of death or re-transplantation.

	(%)
Early re-transplantation	4
Death	28.8
Early death (<3 months post transplantation)	12.8
Causes of death within the first year	
Infection	13.8
Hemorrhagic shock	1
Cardiac disease	3.9
Pulmonary embolism	1
Unknown cause of death	1.9

**TABLE 5:** Factors associated with death, early death, and/or re-transplantation.

	Death	Early death or re-transplantation
Age at SLKTx	NS	NS
BMI	NS	NS
Sex	<0.001	0.088
Transplant rank	NS	0.0359
KT rank	NS	NS
Initial liver disease	NS	NS
Initial kidney disease	NS	NS
MELD score	0.084	0.129
Donor age	NS	NS
Induction	NS	NS
Thymoglobulin use	NS	NS
rIL2 antibody	NS	NS
Creatinine concentration at 1 year	0.0339	NA
ALT levels at 1 year	NS	NA
GGT levels at 1 year	0.0015	NA
Positive cross-match (LCT)	NS	NS
pDSA	NS	NS
Highest pDSA MFI >5,000	NS	NS
Highest pDSA MFI >10,000	0.037	NS
MFI for total pDSA >10,000	0.0498	NS

**TABLE 6:** Multiple regression model for factors involved in patient survival: **(A)** multiple regression model including all factors associated with the risk of death and available at the time of transplantation (*p* < 0.1); **(B)** multiple regression model including all factors associated with an early risk of death (within 3 months post transplantation) or re-transplantation that were present at the time of transplantation (*p* < 0.1).

A- Patient survival according to the pretransplant parameters.				
	Coefficient number	Degrees of freedom	Chi-squared	Prob > Chi^2^
Male/Female	1	1	11.52458	0.0007
Highest pDSA>10,000	1	1	11.91784	0.0006
MELD score at transplantation	1	1	2.03875	0.1533

**B- Early death or re-transplantation.**				

	**Coefficient number**	**Degrees of freedom**	**Chi-squared**	**Prob > Chi^2^**

Male/Female	1	1	4.26763	0.0388
Liver transplant rank	1	1	1.68338	0.1945
MELD score at transplantation	1	1	1.12243	0.2894
Highest pDSA>10,000	1	1	3.33316	0.0679

Based on these data, we performed a multivariate analysis of the risk of death. In the first model, which included the sex of the recipient, highest pDSA, MELD score, serum creatinine concentration at 1 year and GGT level at 1 year, only highest pDSA with a MFI >10,000 tended to be associated with the risk of death (*p* < 0.058) (not shown). When only initial parameters before transplantation were included in the model (sex of the recipient, highest pDSA with a MFI >10,000, and MELD score), both sex (male) and highest pDSA with a MFI >10,000 were identified as independent risk factors for death (*p* < 0.0007) (Table 6).

### Overall graft survival

Death-censored survival was 95% at 1 year, 92% at 3 years, and 85% 5 years post-transplantation for liver grafts and 100, 98, and 98%, respectively, for kidney grafts (Figure 1). Four (7.1%) liver allografts were lost due to vascular complications. Three of the patients concerned underwent re-transplantation (5.2%) and one died from a severe infection before re-transplantation could be performed. None of the liver allografts were lost due to a rejection episode. Two (3.6%) kidney allografts were lost due to the recurrence of oxalate nephropathy, with no sign of acute or chronic rejection on the renal biopsy.

**FIGURE 3:**
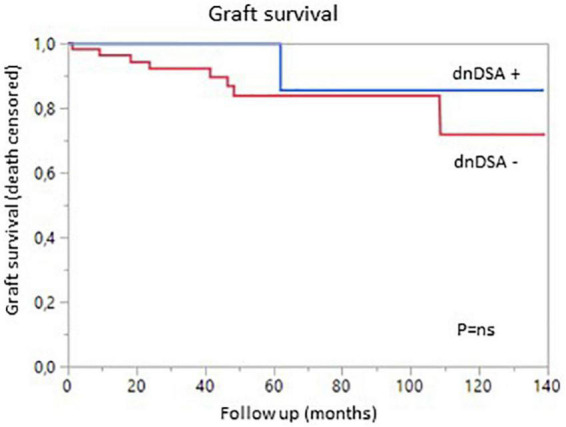
Graft survival (death-censored) according to the presence (blue) or absence (red) of *de novo* DSA.

We then analyzed the impact of pDSA on organ survival. Neither a highest pDSA nor the total MFI for all pDSA was associated with a poorer graft outcome (Figures 2D,E). dnDSA developed in 21.7% of the patients and were mostly directed against MHC class II. The MFI values of dnDSA were between 560 and 3607. The occurrence of dnDSA was not associated with graft loss or patient death (Figure 3).

### Liver and kidney rejection

Liver and/or kidney rejection occurred in six patients (6.1%) (Table 2). The rates were similar in non-sensitized and sensitized patients, and in patients with and without pDSA, for both organs (Table 2). Liver rejection occurred in three patients (2.9%). Two patients (1.9%) developed an acute cellular rejection of the liver graft (Banff 5) and the third (0.9%) suffered chronic liver rejection. No antibody-mediated liver rejection was observed. Kidney rejection occurred in five patients (5.8%), two of whom were pDSA-negative, the other three being pDSA-positive. The rejection in pDSA-negative patients was grade 1a acute cellular rejection of the kidney. In pDSA-positive patients, we observed two cases of antibody-mediated kidney rejection and one case of borderline changes alone in a third patient. No concomitant or sequential rejection of both the liver and kidney grafts was observed.

The overall incidence of borderline changes, ACR, AMR and chronic rejection in liver and kidney allografts was similar in pDSA-negative and pDSA-positive patients (Figure 3). The presence of class I or class II pDSA had no impact on the occurrence of rejection.

### Liver and kidney function

**FIGURE 4:**
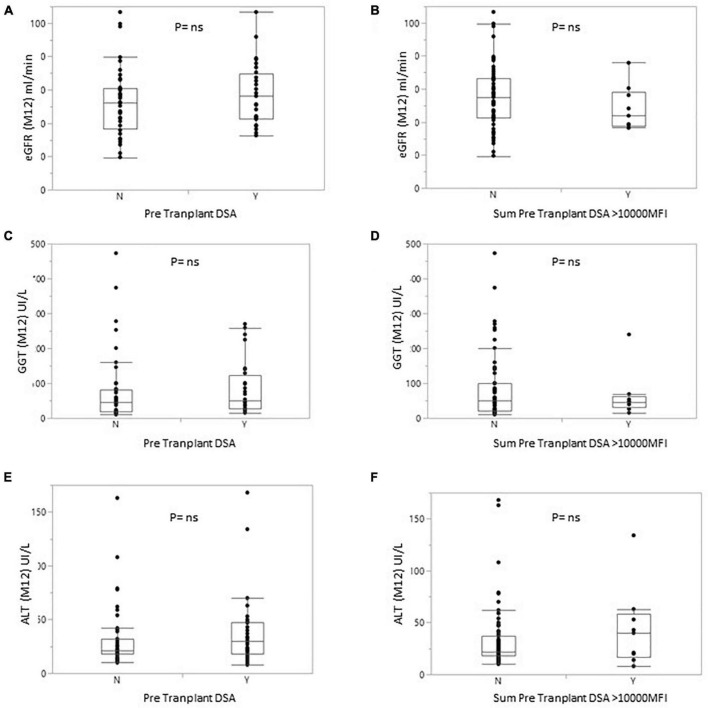
Box plot of eGFR **(A,B)** or GGT **(C,D)** or alanine transaminase **(E,F)** levels for patients with or without pDSA **(A,C,E)** or with a highest pDSA with a MFI >10,000 **(B,D,F)**. Y, yes; N, no.

At 1 year, liver markers and renal function did not differ significantly between non-sensitized patients and patients with pDSA. Serum levels of alanine aminotransferase (ALT) and GGT were 35.95 ± 38.86 and 71.72 ± 100.66 IU/L, respectively, in pDSA-negative patients vs. 39.19 ± 26.69 IU/L (*p* = 0.173) and 82.19 ± 72.58 IU/L (*p* = 0.274) in pDSA-positive patients (Figure 4). Serum total bilirubin concentrations were significantly mildly increased in pDSA-negative patients (11.44 ± 5.01 μmol/L) than in pDSA-positive patients (8.74 ± 4.87 μmol/L; *p* = 0.031) but remained within the normal range (upper limit of the normal range: 17 μmol/L). The mean eGFR was similar in pDSA-negative and pDSA-positive recipients (58 ± 18.1 vs. 49.7 ± 20.6 ml/min/1.73 m^2^; *p* = 0.104). Renal function did not differ between patients with class I pDSA (*p* = 0.631) and those with class II pDSA (*p* = 0.112) or between patients with and without a positive cross-match (*p* = 0.842).

### Impact of treatment

**FIGURE 5:**
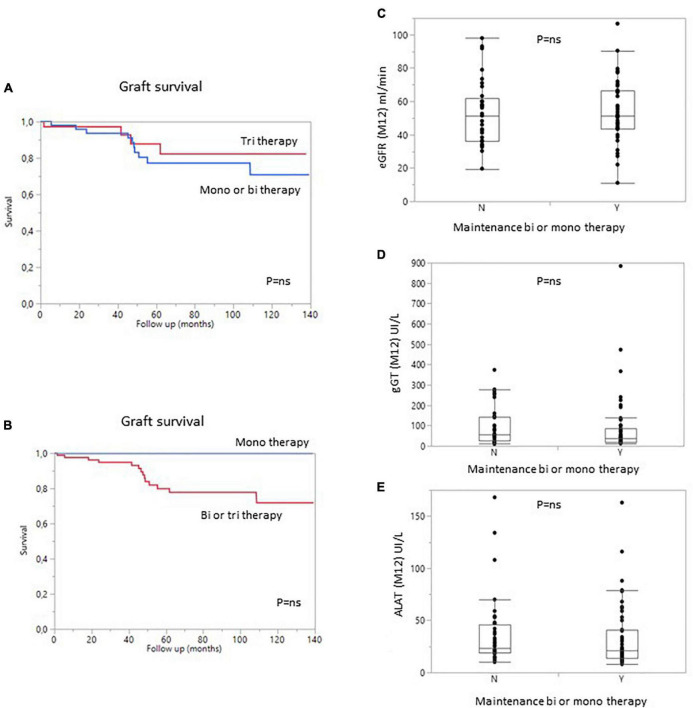
Graft survival (death-censored) according to the number of immunosuppressive drugs in the regimen at 1 year. **(A)** Patients with bi- or monotherapy (blue) vs. triple immunosuppression (red). **(B)** Patients with monotherapy (blue) vs. bitherapy or triple immunosuppression (Red). **(C)** Estimated GFR, **(D)** gamma GT levels, and **(E)** ALAT levels in patients on bi- or monotherapy vs. triple immunosuppression at 1 year. Y, yes; N, no.

The type and intensity of immunosuppression can affect patient and/or graft survival, and a large proportion of patients in this study received maintenance treatment with one or two immunosuppressive agents (57%). We therefore analyzed the impact of treatment dose on patient and graft survival. Surprisingly, the use of a triple regimen was more frequent in sensitized patients than in patients with pDSA or in non-sensitized patients. The level of immunosuppression was not associated with the risk of developing dnDSA. Conversely, the occurrence of dnDSA was not associated with impaired graft survival (Figure 3). The use of a monotherapy or of a two-agent regimen had no impact on patient or graft survival (Figure 5). Consistent with these results, eGFR at months 12 and liver enzyme levels (ALT or GGT) did not differ between patients on triple-IS therapy and patients treated with one or two IS agents. The two-agent regimens mostly used was a combination of steroids and tacrolimus.

## Discussion

Simultaneous kidney and liver transplantation has become more common in the last decade because of an increase in the number of patients with concomitant hepatic and renal failure (24). The increasing use of SLKT has been driven by progress in the management of such transplants, but also by the aging of the population and the chronic renal toxicity of calcineurin inhibitors in patients who have undergone liver transplantation (20, 25, 26). Graft and patient outcomes have improved. We and others have reported a lower risk of acute rejection in SLKT, which has led to strategies for minimizing immunosuppressive treatment in this context (1). The development of new techniques, such as Luminex assays, for identifying and quantifying pDSA and/or dnDSA and improving the description of AMR, has led to better characterization of the status of sensitized patients and improvements in AMR diagnosis in kidney transplantation. The role of DSA following liver transplantation remains a matter of debate, but a few reports have highlighted the risk of graft loss in cases of a positive cross-match and the presence of class II pDSA (2).

We describe here our large experience in SLKT. More than half of our patients had anti-HLA antibodies, and more than 40% had pDSA at the time of SLKT, a figure higher than reported in previous studies (21, 27, 28). Our findings confirm that the risk of developing acute low-grade rejection is low in SLKT, consistent with previous reports of lower rates of acute cellular rejection in SLKT patients than in kidney transplant patients. Moreover, we found that the rates of both cellular and AMR were similar in patients with and without preformed DSA, for both organs, and the presence of pDSA being associated with a higher risk of death. We also found that the minimization of the maintained immunosuppression could be offered to some patients with pDSA with no deleterious effects on liver and kidney graft function.

Like Del Bello et al. and Taner et al., we found no evidence of a higher risk of acute rejection in patients with pDSA, or even in those with a positive cross-match at the time of SLKT (17, 28). However, other studies have reported a higher risk of AMR in cases of pre-formed class II antibodies or positive cross-matches (19, 21, 29). The differences between studies may be explained by the more aggressive immunosuppression for induction in our cohort, based principally on the use of a combination of polyclonal antibodies and IVIG.

We found that the risk of death was higher in male patients, in patients with high titers of highest pDSA and in patients with a total MFI for all pDSA exceeding 10,000 MFI. There was also a trend toward a higher risk of death in patients with higher MELD scores. Furthermore, the risk of death was higher in patients with high serum creatinine or GGT levels 1 year after transplantation, without any signs of acute rejection. In a multiple regression model including only parameters available at the time of transplantation, only the sex of the patient and highest pDSA with a MFI >10,000 were associated with the risk of death. This finding is consistent with previous reports indicating that the risk of death is higher in sensitized patients (28, 29). The risk of death was associated with a MFI for pDSA exceeding 5,000 in these previous studies. The difference between this value and the value identified in our study (pDSA MFI >10,000) may reflect differences in the Luminex techniques used or in the type of immunosuppressive treatment. However, both our results and these previous findings suggest that significant immunization is associated with a higher risk of death. By contrast, we surprisingly found no correlation between the risk of patient death and positive cross-match, even though positive cross-matching is associated with high levels of pDSA. This result can be explained by a lack of power due to the small number of transplant patients with a positive cross-match included. We cannot exclude that some of dead patients had developed acute humoral rejection and biopsies required for the diagnosis of rejection were not performed because of the severity of the situation. Therefore, we suggested that SLKT has to be strongly evaluated in case of pDSA >10,000 MFI because of a possible risk and/or because the use of more aggressive initial immunotherapy which can be associated with a higher risk of death due to graft dysfunction or immunosuppressive complications. As previously reported, most deaths after SLKT were due to infectious diseases or cardiovascular events. We found that the risk of early death or re-transplantation was associated with the rank of the liver transplant, with a trend for these events to be more frequent in male patients and in patients with a high MELD score, as described for liver transplantation. In a multiple regression model that also included pDSA, the risk of death or re-transplantation was associated with sex (higher risk for men) and tended to be associated with a highest pDSA with a MFI exceeding 10,000. These results suggest that SLKT should be carefully assessed in cases of high MFI for the highest pDSA or if the sum of MFI for all pDSA exceeds 10,000.

Given the lower incidence of rejection for both kidney and liver grafts and the risk of death due to infectious diseases in SLKT patients (5), we decided to minimize the maintenance immunosuppressive treatment. One year after transplantation, 57% of patients were treated with only one (mostly CNI) or two (mostly low dose steroids plus CNI) agents. In cases of triple maintenance therapy, the patients also received a low dose of mycophenolate mofetil. Interestingly, 80% of the non-sensitized patients and the patients with pDSA were on mono- or dual therapy at 1 year, due to the large decrease in pDSA levels post transplantation (at 1 month), leading physicians to reduce immunosuppressive treatment. We also found that the minimization of immunosuppression had no effect on the survival of patients or grafts. In addition, liver test results and eGFR were similar for patients with minimized immunosuppression and those on the triple regimen, even for the subgroup of patients with pDSA or sensitized patients. This finding is consistent with the results published by Kamal et al. showing patient and graft outcomes for patients on maintenance therapies consisting of CNI and steroids. By contrast, as reported by Del Bello et al., the frequency of dnDSA was similar to that observed for kidney or LTA (10, 28, 30, 31). More than 20% of our patients developed dnDSA, mostly against class II. This finding is consistent with previous reports (28, 31). However, titers remained low, as less than 7% of patients had a MFI over 1,000 and the maximum MFI was below 4,000. The presence of pDSA was not associated with the occurrence of acute clinical rejection. These results suggest that, in the context of SLKT, maintenance therapy can be minimized, even in sensitized patients.

Our study has several limitations, due to its retrospective monocentric design. The patients were managed in a generally homogeneous manner, but some differences emerged during the minimization of immunosuppression in immunized patients. In addition, protocol biopsies were not routinely performed to analyze the incidence of subclinical rejection that might have been associated with the further development of graft dysfunction. In addition, we assessed antibodies directed against A/B/DR/DQ, but not those directed against DP or Cw, because the donors were in a large majority not genotyped for these alleles.

## Conclusion

In conclusion, our results indicate that pDSA were associated with a higher risk of mortality in SLKT patients, but not with kidney or liver dysfunction. The minimization of maintenance immunosuppression can be proposed, even for patients with pDSA. Further prospective studies are required to determine the optimal induction therapy for these patients. Our findings suggest that SLKT is not recommended in cases of high pDSA titers, and further studies are required to determine the optimal induction and maintenance therapy for these patients.

## Data availability statement

The original contributions presented in this study are included in the article/supplementary material, further inquiries can be directed to the corresponding author.

## Ethics statement

This study was approved by the Institutional Review Board of Paris-Sud University. Written informed consent for participation was not required for this study in accordance with the national legislation and the institutional requirements.

## Author contributions

FS and AD drafted and wrote the manuscript. J-LT was responsible for HLA phenotyping and anti-HLA antibody characterization. MD and FH were responsible for data collection and biostatistics. ME, PI, MBo, AC, AS, FS, and AD were involved in patient care. All authors reviewed and approved the final version of the manuscript.

## Abbreviations

ADCC: antibody-dependent cell-mediated cytotoxicity
ALT: alanine aminotransferase
ATG: antithymoglobulins
CDC: complement-dependent cytotoxicity
CKD-EPI: chronic kidney disease epidemiology
dnDSA: *de novo* donor-specific antibody
DSA: donor-specific antibody
eGFR: estimated glomerular filtration rate
gGT: gamma glutamine transferase
HLA: human leukocyte antigen
IS: immunosuppression
LTA: liver transplantation alone
MELD: Model for End-Stage Liver Disease
MFI: mean fluorescence intensity
pDSA: preformed donor-specific antibody
SLKT: simultaneous liver–kidney transplantation.
